# MRI Investigation of Kidneys, Ureters and Urinary Bladder in Rabbits

**DOI:** 10.3390/vetsci11110575

**Published:** 2024-11-16

**Authors:** Rosen Dimitrov, Kamelia Stamatova-Yovcheva, Georgi Georgiev

**Affiliations:** 1 Department of Veterinary Anatomy, Histology and Embryology, Faculty of Veterinary Medicine, Trakia University, 6000 Stara Zagora, Bulgaria; rdimitrov288@gmail.com; 2 Department Veterinary Anatomy, Physiology, and Animal Sciences, Faculty of Veterinary Medicine, University of Forestry, 1000 Sofia, Bulgaria; gigeorgiev@ltu.bg

**Keywords:** rabbit, MRI, kidneys, ureters, urinary bladder, anatomy

## Abstract

Twelve clinically healthy and sexually mature New Zealand White rabbits were studied. The non-contrast imaging included T1-weighted and T2-weighted spin-echo and gradient-echo sequences in the transverse, sagittal and dorsal planes. Transverse MRI (T2-weighted image) through L1 demonstrated only the right kidney. The transverse T2-weighted image through L2 showed both kidneys. The cranial part of the urinary bladder on T1-weighted transverse scans through L4 was flexed to the left. The T2-weighted sagittal image 30 mm to the right of the median plane showed the right kidney, the right ureter and the urinary bladder. The T2-weighted sagittal image 30 mm to the left of the median plane showed part of the left kidney, the left ureter and the urinary bladder. The T2-weighted sagittal image 45 mm to the left of the median plane presented the lateral part of the left kidney. The dorsal MRI image (T2-weighted sequence) through the horizontal plane 30 mm ventral to the spine demonstrated the whole organs. These data will be useful in imaging anatomy and diagnostic studies of various pathologies of the excretory system in rabbits and other mammalian species.

## Simple Summary

The presented MRI results increase the imaging morphologic knowledge of the kidney, ureter and urinary bladder. The data will be useful in imaging anatomy and diagnostic studies of various pathologies of the excretory system in rabbits and other mammalian species. MR urography is a modern imaging technique for the examination of the entire urinary tract that replaces the gold standard excretory urography for the detection of urinary pathological changes.

## 1. Introduction

The rabbit kidney is unipapillary, with renal pelvis recesses (dorsal and ventral) in the medullary part. Thus far, the rabbit is the only known mammal whose renal tubules may be separated on kidney MR slices while their epithelium remains intact; therefore, it may be used in the in vitro studies of kidney morphology. The right kidney can be palpated in the thoracolumbar region and is located cranially to the left one. In some individuals, the adipose capsule is thickened, causing ventral displacement of the kidneys. The thin-walled urinary bladder is situated in the ventrocaudal part of the abdominal cavity [1,2].

The excretory radiography of the normal urinary tract provides anatomotopographical and functional data on the kidneys, ureters and urinary bladder of New Zealand White rabbits. It is established that the right kidney is located between the transverse planes from T13 to L2 vertebrae, while the left one is between L2 and L4. Anatomical landmarks for the evaluation of morphotopographical features of the kidneys and urinary bladder in rabbits are their association with the length and localization of the second and fifth lumbar vertebrae [3,4].

In rabbits, diseases affecting the kidneys are widespread (renal agenesis, asymptomatic congenital kidney cysts, bacterial infections, renal abscesses, staphylococcal nephritis, pyelonephritis, renal amyloidosis, urolithiasis and tumors). The application of the imaging methods for their diagnosis is available. Normal rabbit kidneys are visualized. In healthy rabbits, the imaging methods provide detailed images of kidney parenchyma and the urinary tract [5,6,7].

The rabbit is commonly used as an animal species in laboratory research and is approved as a suitable experimental model in kidney research with respect to human donor kidney transplantation. The imaging methods of kidneys in healthy rabbits provide topographical, morphometric and radiographic data on kidney morphology. For ultrasound examination, the animal is positioned in both supine and lateral recumbency. The kidneys are examined in the longitudinal and transverse planes. In the longitudinal plane, hypoechoic oval areas (renal pelvis recesses) are observed within the kidney medulla, separated by hyperechoic peripelvic columns, and the kidney papilla is hyperechoic. For a computed tomography (CT) exam without a contrast medium, the rabbits are in ventral recumbency. The right kidney is slightly cranial to the left one by its length and separated from the stomach by the right liver lobe. Both kidneys appear as dense, soft tissue findings with distinct borders in relation to the adjacent organs [8].

MRI of the anatomical and physiological features of kidneys was used for obtaining anatomic images, monitoring renal function and evaluating the anatomic interrelationships of the macroscopic compartments of kidneys (cortex, medulla, sinus). MRI allows for a relatively high-quality contrast and defined tissue and organ imaging in comparison to CT. The multiplanar MRI image acquisition is not associated with body exposure to ionized radiation. MRI presents relatively precise data regarding the staging of human kidney tumors [9,10,11,12].

Rabbits are used as animal models in human medical urological research studies. They are excellent laboratory biological models, as they are easy to handle, not expensive and with well-acknowledged pathophysiological characteristics. Therefore, anatomical studies of rabbits are an important part of modern experimental medicine [13,14,15].

Spin-echo T1-weighted MRI demonstrates the corticomedullary differentiation of normal human kidneys. Images of the kidney cortex are relatively hyperintense than those of the medulla due to the larger amount of extracellular fluid in the latter. The lack of corticomedullary differentiation on T1-weighted images is a non-specific sign of kidney pathology. The rat is a biological model for the investigation of human kidneys. In this animal species, the kidney cortical and medullary MR structures are comparable to human ones. The hydration status of rats influences the intensity of cortical and medullary MR signals. The structure of the renal cortex and the inner medulla are similar to those of men. The rat kidney possesses two additional layers that are located between the cortex and the inner medulla: the outer and inner areas of the outer medulla. The outer area is visualized with the highest signal intensity on T1-weighted images but with a lower signal intensity on T2-weighted scans. The inner area of the outer medulla is hypointense on T1-weighted scans compared to the other medullary layers and exhibits a relatively higher signal intensity on T2-weighted scans. The differences in human and rat kidneys are related to MR imaging of the corticomedullary junction. The differentiation between the rat renal cortex and medulla is similar to that of men, only following the hydration of examined rats. Therefore, the lack of differentiation between the cortex and medulla on T1-weighted MRI images is a non-specific finding in men, presumably due to different kidney diseases or hydration of the patient [16].

MRI provides information about the kidneys and ureters through spatial anatomic resolution. The MRI protocol combines morphological and functional evaluations of kidneys without nephrotoxicity. The only disadvantage of MRI in comparison to CT is the lower quality of uroliths’ image. In men, MRI is performed in dorsal recumbency with flexed stifle joints. MRI examination is performed in consequent dorsal planes with phase-encoding from the left to the right. It provides exact data on the corticomedullary differentiation of renal parenchyma on T1-weighted scans. The normal renal fibrous and adipose capsules are identified as relatively hard. The fibrous capsule is remarkably visualized in inflammations of the kidney parenchyma [17,18].

MR urography is a modern imaging technique used for the examination of the entire urinary tract. The transverse MRI of urinary organs has replaced excretory urography as the gold standard for the detection of urinary pathological changes in clinical practice [19,20,21,22,23].

On T1-weighted scans of human kidney parenchyma, the hyperintense cortical area is distinguished from the hypointense medulla. On T2-weighted scans, the distinction of both areas is less pronounced, and the cortex appears hypointense against the medulla. The fibrous capsule is imaged as a hypointense thin linear finding on both T1- and T2-weighted sequences [24].

T1-weighted MR of normal kidneys in chinchillas provides images with anatomical contrast. The kidney findings are more hypointense than peripheral soft tissues. The renal cortex, medulla and pelvis are clearly visualized. The right kidney image is relatively more hyperintense than that of the left kidney. On T2-weighted scans, the renal cortex appears relatively more hypointense than the hyperintense medulla. Among kidney structures, the signal of the renal pelvis is relatively the most intense [25,26].

Two MRI protocols are acknowledged—static and dynamic. The static protocol, also known as uro-resonance in T2 (hydrography), employs T2-weighted sequences with a long relaxation time for urine. The latter allows obtaining a hyperintense image of the urinary tract. Static uro-resonance is efficient for investigations of kidney-collecting systems. This protocol does not require an application of contrast. With the dynamic protocol, contrast medium is applied to evaluate excretory renal function [27,28].

The lack of sufficient data regarding the visualization of rabbit kidney parenchyma and the collecting system, ureters and urinary bladder is the main objective of this present study. MRI is an innovative method for investigating the imaging anatomy of these organs for diagnostics and therapy for diseases and abnormalities in this region.

## 2. Materials and Methods

### 2.1. Materials

Twelve New Zealand White rabbits were included in this study. The animals were clinically healthy and sexually mature (8 months of age), weighing 2.8–3.2 kg. The current study has been approved by permit for the use of animals in experiments No. 377, issued by the Ethics Committee of the Ministry of Agriculture and Food, Bulgarian Animal Safety Agency, with grant no. 293 of 29.02.2024, Sofia, Bulgaria, in compliance with the provisions of the Animal Protection Act in Bulgaria (promulgated in State Gazette No 13/8 February 2008) and the European Convention for the Protection of Vertebrate Animals Used for Experimental and Other Scientific Purposes (ETS No 123, OJ L 222, 24 August 1999, pp. 31–37). Food was withdrawn 4 to 6 h prior to the MRI. Good body hydration status was essential for obtaining precise MRI data of the urinary organs. The rabbits were housed at 25 °C under a 12:12 h light/dark cycle. The contrast resolution of the images used is better for the morphological differentiation of particular soft tissue structures, including the urinary system [29,30,31,32,33,34].

MRI was performed using a tunnel MR scanner (1.5 T, Magnetom Essenza, version of software—Tim + Dot, Siemens Healthcare, USA, Whole body imaging; Dot^®^, The Siemens MRI software, Ferndale, MI 48220). The animals were positioned horizontally in the supine position on a flat table, as the lumbar region was positioned in the isocenter of the magnet. Examinations were performed with a superconductive magnet at 1.5 T using surface coils and r-weighted spin-echo sequences (time to repeat/time to echo (TR/TE) = 450/20, matrix = 256 and between 4–6 averages). Dorsal, sagittal and transversal scans were conducted with a slice thickness of 2 mm.

### 2.2. Methods

#### 2.2.1. Anesthetic Protocol

The rabbits were anesthetized with 15 mg/kg Zoletil^®^ 50 (Virbac, Carros, France) (IM) (tiletamine hydrochloride 125 mg and zolazepam hydrochloride 125 mg in 5 mL of the solution) (Virbac, France). Anesthesia was potentiated with a Ketaminol^®^ 10 solution (Intervet, Unterschleißheim, Germany) (IM) (Ketamine hydrochloride 100 mg/mL and Benzethonium chloride 0.1 mg/mL), in a dose of 0.5 mL/kg [30,31].

The rabbits were examined with caudally extended hip joints and flexed stifle joints. Immediately before the examination, a spasmolytic drug (Ketonal, Lek Pharmaceuticals d.d., Verovškova 57, Ljubljana, Slovenia, 100 mg/2 mL solution for injection, 1 mg/kg i.m.) was applied to block intestinal peristalsis and diaphragmatic breathing, which may deteriorate the image quality produced by the 1.5 Tesla MR scanner [35,36].

#### 2.2.2. Anatomical Landmarks

Transverse abdominal sections were obtained between the planes through the 10th thoracic and the last lumbar vertebrae. Sagittal sections were performed in planes located at 15, 30 and 45 mm to the left and to the right of the median plane. The dorsal sections were obtained in planes that were ventral to the spine at distances of 15, 30, 45 and 60 mm [30,31].

The non-contrast imaging included axial T1-weighted and T2-weighted spin-echo and gradient-echo sequences in the transverse, sagittal and dorsal planes [26,30,31].

MRI slices were aligned on the following bones and soft tissue objects: for bone anatomical landmarks, these were on transverse sections, depending on the topography of the slices to the corresponding vertebra; on dorsal sections, depending on the topography of the slices to the spine; and on sagittal sections, depending on the topography of the slices to the median plane. The soft tissue anatomical landmarks on transverse, dorsal and sagittal sections were the liver, stomach, small and large intestines, mesenterial root, mesentery, adipose tissue, diaphragm and abdominal wall [30,31].

#### 2.2.3. MRI Algorithm

This study was performed under the following research imaging protocol: a magnetic field strength of 1.5 T; a superconducting type magnet; 70 cm diameter of the magnetic cylinder; 4 Channel Special-Purpose coil elements; a matrix of 256 × 256; pixels were 1 mm^2^ transversal, sagittal and dorsal anatomical images weighted in T1 (TE 120 ms, flip angle 90°), and T2 spin-echo and gradient-echo sequences (TR 2000 ms, TE 100 ms, flip angle 90°); 2D acquisition schemes were applied for the sequences; the FOV was 50 cm^3^ (with mean values of 250/250) in all directions; the SNR was 20 dB; the echo time (TE) was 14 ms for T1 and 90 ms for T2; the repetition time (TR) was 500 ms for T1 and 4000 ms for T2; the voxel size was 10 mm^3^; the abdomen and pelvis were scanned with a full urinary bladder. The size of the voxel was increased to identify the number of tissue components of the organs. At the same time, the number of nuclei increased parallel to the SNR [12,30,31,34].

MR urography was performed in the presence of static fluid in the collecting part of the urinary organs to obtain a panoramic view of the entire urinary collecting system without the application of intravenous contrast [37].

The images used were collected adequately and precisely from all twelve studied animals. The highest quality ones are presented.

## 3. Results

### 3.1. Transverse MRI

**Figure 1:**
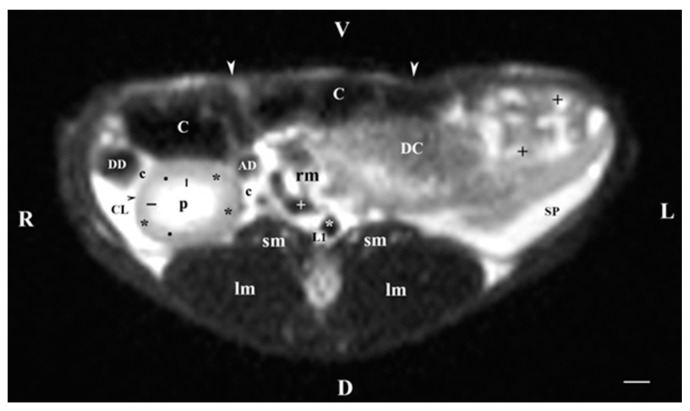
MRI imaging anatomy of organs from the middle abdominal region (transverse section at the L1 level); R-right; L-left; D-dorsal; V-ventral; (T2-weighted sequence). Note: (C) caecum; (DC) descending colon; (black cross) jejunum; (SP) spleen; (lm) longissimus muscle; (sm) psoas muscles; (rm) root of mesentery; (white star) abdominal aorta; (white cross) caudal vena cava; (black star) renal cortex; (black horizontal arrows) renal medulla; (black line) renal medulla; (black point) corticomedullary junction; (c) renal adipose capsule; (CL) caudate lobe; (DD) descending duodenum; (AD) ascending duodenum; (white perpendicular arrow) abdominal wall. Line = 10 mm.

The transverse MRI (T2-weighted sequence) of organs at the level of the first lumbar vertebra (L1) visualized only the signal characteristics of the right kidney (Figure 1). The latter had an oval, dorsoventrally flattened shape. The renal pelvis was hyperintense and oval. Its borders with the inner part of the medulla were wavy due to the presence of kidney recesses and the irregular relief of crista renalis. The inner part of the medulla was hyperintense compared to the relatively hypointense image of the outer medulla. The corticomedullary definition was sharply distinguished and outlined the transition between the hyperintense medulla and the relatively hypointense cortex. The fibrous capsule of the right kidney appeared as a thin, linear finding. Its intensity was intermediate compared to the hypointense cortex and the hyperintense renal adipose capsule. Peripherally and on the right, the right kidney was separated from the hypointense ventrolaterally located *pars descendens duodeni* by the adipose capsule. The latter touched the caudate lobe of the liver (Figure 1). Ventromedial to the right kidney was the hypointense image of the ascending duodenum. Dorsal to the right kidney was the hypointense signals of the psoas muscles and longissimus muscles. The left kidney image was not visible to the left (Figure 1).

**Figure 2:**
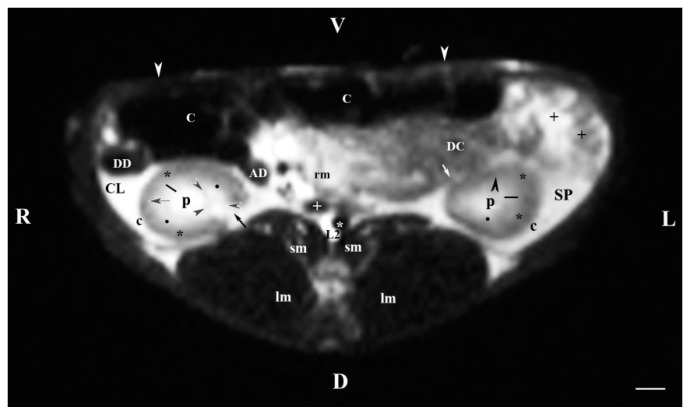
MRI imaging anatomy of organs from the middle abdominal region (transverse section at the L2 level); R-right; L-left; D-dorsal; V-ventral; (T2-weighted sequence). Note: (C) caecum; (DC) descending colon; (black cross) jejunum; (SP) spleen; (lm) longissimus muscle; (sm) psoas muscles; (rm) root of mesentery; (white star) abdominal aorta; (white cross) caudal vena cava; (black star) renal cortex; (p) renal pelvis; (black horizontal arrow and black point) right renal corticomedullary junction; (black perpendicular arrowhead) external medullary part; (white oblique arrow) commencement of the left ureter; (CL) caudate lobe; (DD) descending duodenum; (AD) ascending duodenum; (c) adipose capsule; (oblique black arrowhead) dorsal and ventral renal recess; (oblique black arrow) renal hilus and commencement of the right ureter; (white perpendicular arrowhead) abdominal wall. Line = 10 mm.

The transverse MRI (T2-weighted sequence) through L2 showed both kidneys: the cranial part of the left one and the middle part of the right one. The hyperintense hilus of the right kidney with the beginning of the ureter, dorsolateral to the hypointense signal of the ascending duodenum, was seen dorsomedially (Figure 2). The descending colon was visualized ventrally to the beginning of the left ureter. The dorsal and ventral kidney recesses appeared as hypointense oval findings between the kidney pelvis and the medulla. The corticomedullary definition at the left kidney was well represented. The left kidney image had an almost triangular transverse profile, surrounded by the hyperintense adipose capsule separating it from the spleen. The proximal end of the ureter was seen as a hypointense tubular finding at the ventromedial border of the left kidney. The distance between images of the left kidney and medially located hypointense image of the abdominal aorta was significantly greater than the distance from the right kidney and medially located hypointense caudal vena cava (Figure 2). The descending colon and jejunum with mesentery and cecum were visualized ventral to the left kidney. The difference in signals of the left kidney’s inner and outer medullary parts was similar to the pattern of the right kidney. The fibrous capsule of the left kidney appeared as a thickened linear hypointense finding in comparison with the capsule of the contralateral kidney (Figure 2).

**Figure 3:**
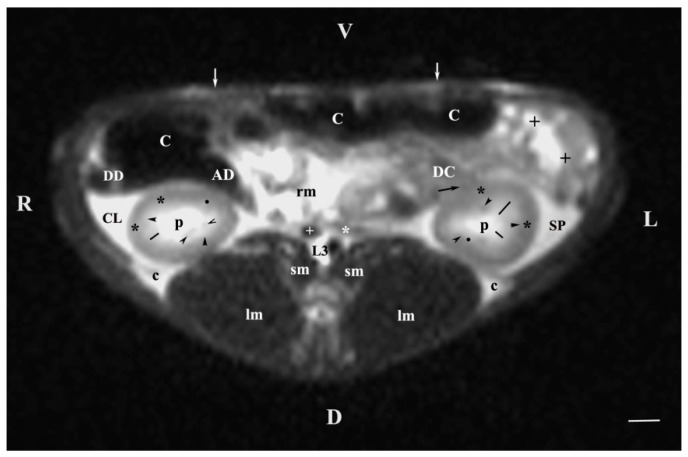
MRI imaging anatomy of organs from the middle abdominal region (transverse section at the L3 level); R-right; L-left; D-dorsal; V-ventral; (T2-weighted sequence). Note: (C) caecum; (DC) descending colon; (black cross) jejunum; (SP) spleen; (lm) longissimus muscle; (sm) psoas muscles; (rm) root of mesenterium; (white star) abdominal aorta; (white cross) caudal vena cava; (black star) renal cortex; (p) renal pelvis; (black horizontal arrowhead) external medullary part (black point) renal corticomedullar junction; (black line) renal medulla; (oblique black arrowhead) dorsal and ventral renal recesses; (black oblique arrow) commencement of the left ureter; (CL) caudate lobe; (DD) descending duodenum; (AD) ascending duodenum; (c) adipose capsule; (white perpendicular arrow) abdominal wall. Line = 10 mm.

The transverse section of the middle abdominal region (T2-weighted sequences) at L3 demonstrated the caudal part of the right kidney and the middle part of the left kidney. The right kidney shape was oval and dorsoventrally flattened, whereas the left kidney was pyramidal. The hyperintense image of the left renal pelvis was also almost triangular, while that of the right one remained oval. The contact between the lateral part of the right kidney and the caudate lobe of the liver was outlined by the hypointense linear image of the fibrous capsule. The anatomical relation between the lateral part of the left kidney and the spleen was also well-defined by the hypointense linear fibrous capsule. The descending duodenum was ventral to the right kidney and the liver. The descending colon remained ventromedial to the left kidney. Hypointense signals of the psoas muscles and longissimus muscles appeared dorsally to both kidneys (Figure 3).

**Figure 4:**
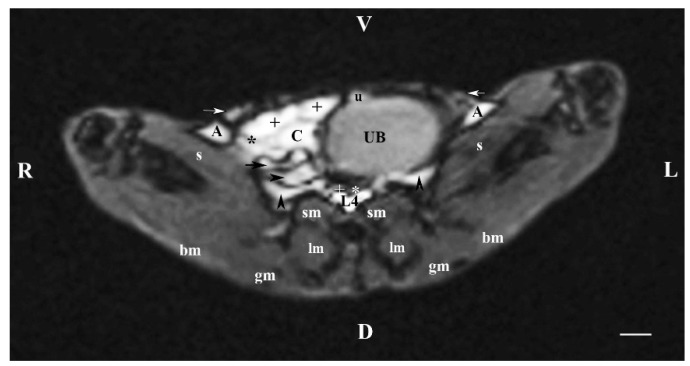
MRI imaging anatomy of organs from the middle abdominal region (transverse section at the L4 level); R-right; L-left; D-dorsal; V-ventral; (T1-weighted sequence). Note: (UB) urinary bladder; (+) jejunum; (*) mesenterium; (C) caecum; (black horizontal arrow) ileum; (black horizontal arrowhead) ascending colon; (black vertical arrowhead) duodenum; (A) adipose tissue; (s) sartorius muscle; (sm) psoas muscles; (lm) longissimus muscle; (gm) gluteus muscle; (bm) biceps femoral muscle; (white horizontal arrows) abdominal wall; (white cross) caudal vena cava; (white star) abdominal aorta; (u) urachus and median ligament of urinary bladder. Line = 10 mm.

In the transverse plane through L4 (T1-weighted sequence), the filled cranial part of the urinary bladder demonstrated flexion to the left with localization of the organ in the left caudal abdomen with the exception of the bladder neck. Therefore, the other abdominal organs in this part of the abdomen were displaced to its right part. Dorsally, only the hyperintense images of the duodenal parts were situated. Cranially, a part of the urachus was seen, again hyperintense vs. the urinary bladder. The urinary bladder image was dorsoventrally flattened and oval, with heterogeneous and relatively hypointense lumen. The T1-weighted sequence of the urinary bladder distinctly defined its borders from the adjacent soft tissues. The lumen of the studied organ was relatively hyperintense vs. its hypointense wall. In this section, ureter findings were not presented (Figure 4).

**Figure 5:**
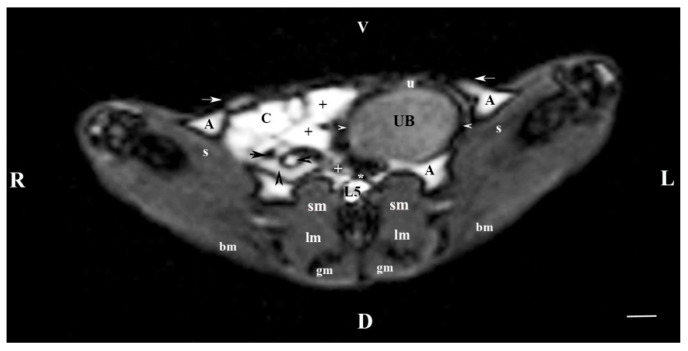
MRI imaging anatomy of organs from the middle abdominal region (transverse section at the L5 level); R-right; L-left; D-dorsal; V-ventral; (T1-weighted sequence). Note: (UB) urinary bladder; (white horizontal arrowhead) urinary bladder wall; (+) jejunum with mesenterium; (C) caecum; (black horizontal arrow) ileum; (black horizontal arrowhead) ascending colon; (black vertical arrowhead) descending duodenum; (A) adipose tissue; (s) sartorius muscle; (sm) psoas muscles; (lm) longissimus muscle; (gm) gluteus muscle; (bm) biceps femoral muscle; (white horizontal arrows) abdominal wall; (white cross) caudal vena cava; (white star) abdominal aorta; (u) urachus and median ligament of urinary bladder. Line = 10 mm.

The transverse section of the abdominal cavity through L5 (T1-weighted sequence) showed a heterogeneous hypointense image of the urinary bladder body compared to the images of the other peripheral organs. The bladder wall was observed as a clear hypointense band with oval borders. The oval and dorsoventrally flattened urinary bladder was visualized in the left caudal part of the abdominal cavity. The hypointense image of the ventral median ligament of the urinary bladder appeared cranioventrally to the bladder body (Figure 5).

**Figure 6:**
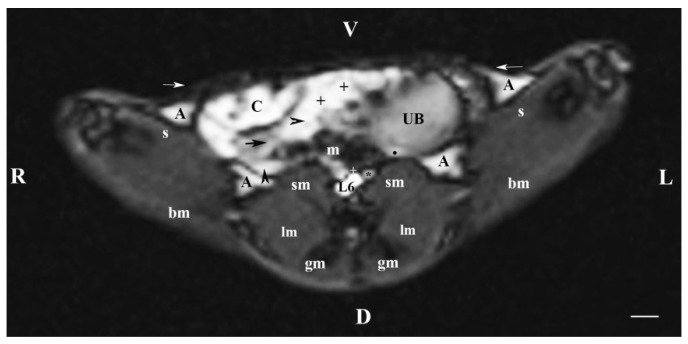
MRI imaging anatomy of organs from the middle abdominal region (transverse section at the L6 level); R-right; L-left; D-dorsal; V-ventral; (T1-weighted sequence). Note: (UB) urinary bladder; (black point) infusion of ureters; (+) jejunum with mesenterium; (C) caecum; (black horizontal arrow) ileum; (black horizontal arrowhead) ascending colon; (black vertical arrowhead) descending duodenum; (A) adipose tissue; (s) sartorius muscle; (sm) psoas muscles; (lm) longissimus muscle; (gm) gluteus muscle; (bm) biceps femoral muscle; (white horizontal arrows) abdominal wall; (white cross) caudal vena cava; (black star) abdominal aorta; (m) mesenterium. Line = 10 mm.

On the transverse section through L6 (T1-weighted sequence), the caudal part of the urinary bladder was located in the left part of the caudal abdomen. The bladder lumen was relatively hyperintense in relation to its wall, which appeared as a thickened linear hypointense finding. Dorsomedial to the urinary bladder, a hyperintense longitudinally oval finding shows the entry of ureters in the bladder wall. The urinary bladder neck was not visible (Figure 6).

### 3.2. Sagittal MRI

**Figure 7:**
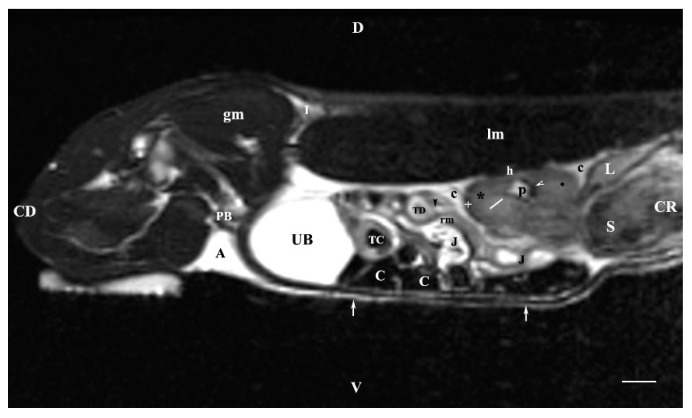
MRI imaging anatomy of organs from the middle abdominal region (sagittal section) (T2-weighted sequence 30 mm to the right of the median plane) (Right kidney). Note: (CD) caudal; (CC) cranial; (D) dorsal; (V)ventral; (UB) urinary bladder; (p) renal pelvis; (black star) renal cortex; (white line) renal medulla; (black point) corticomedullary junction; (h) renal hilus; (white cross) fibrous capsule; (oblique white arrowhead) dorsal and ventral renal recess; (perpendicular black arrowhead) ureter; (S) stomach; (L) liver; (c) renal adipose capsule; (rm) mesenterial root; (J) jejunum; (TD) transverse duodenum; (TC) transverse colon; (C) caecum; (A) adipose tissue; (PB) pubic bone; (lm) longissimus muscle; (gm) gluteal muscles; (I) ilium (white perpendicular arrow) abdominal wall. Line = 10 mm.

A sagittal T2-weighted image of the abdominal cavity 30 mm to the right of the median plane showed almost the entire right kidney (in the dorsal lumbar area), the right ureter and the urinary bladder, as well. The right kidney demonstrated its specific bean shape, and the renal hilus was seen as a hypointense dorsomedially located structure. The renal pelvis was clearly outlined as a hyperintense finding of irregular borders. Peripherally to it, the hypointense dorsal and ventral recesses were visualized. The corticomedullary definition was clearly demarcated as an intense irregular finding of transient intensity situated between the hyperintense medulla and the relatively hypointense cortex. The renal adipose capsule was hyperintense, and the fibrous capsule was hypointense. The craniolateral border of the right kidney touched the heterogeneous hypo signal liver. The inner and outer areas of the kidney medulla were not distinguished. Caudoventrally to the hyperintense adipose capsule of the right kidney appeared the hyperintense tubular right ureter, whose lumen was more intense than its walls. The hyperintense signal of the urinary bladder body was observed in the caudal abdomen, clearly delineated from the adjacent soft tissues (Figure 7).

**Figure 8:**
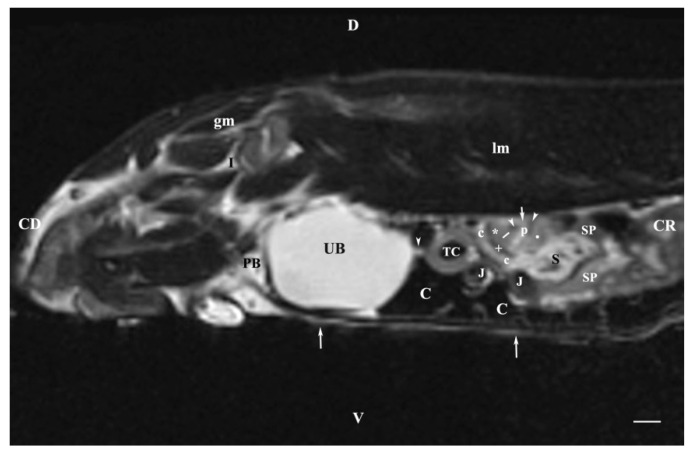
MRI imaging anatomy of organs from the middle abdominal region (sagittal section) (T2-weighted sequence 30 mm to the left of the median plane) (Left kidney). Note: (CD) caudal; (CC) cranial; (D) dorsal; (V) ventral; (UB) urinary bladder; (p) renal pelvis; (white star) renal cortex; (white line) renal medulla; (white point) corticomedullary junction; (perpendicular white arrow down directed) renal hilus; (oblique white arrowhead) renal recesses; (white star) renal cortex; (perpendicular white arrowhead) ureter; (S) stomach; (SP) spleen; (c) renal adipose capsule; (white cross) fibrous capsule; (J) jejunum; (TC) transverse colon; (C) caecum; (PB) pubic bone; (I) ilium; (lm) longissimus muscle; (gm) gluteal muscle; (I) ilium; (white perpendicular arrow upward directed) abdominal wall. Line = 10 mm.

The sagittal T2-weighted image obtained 30 mm to the left of the median plane demonstrated the incomplete image of the left kidney (the lateral part) located in the lumbar area, the left ureter and the urinary bladder. The adipose capsule of the left kidney was clearly defined and hyperintense. The fibrous capsule was linear, hypointense and with irregular outer and inner borders. The renal pelvis signal was hyperintense with indistinct peripheral outlines. The transition between the inner and outer medulla was not observed. The corticomedullary definition was marked. Cranioventrally, the left kidney communicates with the spleen and the stomach. The left ureter was visualized as a hyperintense tubular finding in the segment between the transverse colon and the urinary bladder. The bladder lumen appeared relatively more hypointense than its hyperintense wall. It was located in the caudal abdomen, and was with irregular shape and indistinct borders (Figure 8).

**Figure 9:**
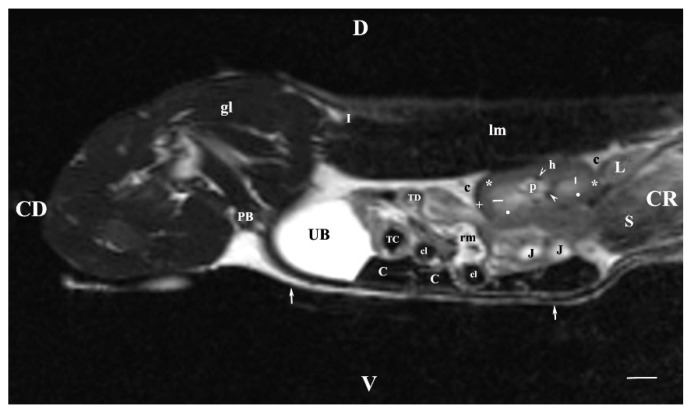
MRI imaging anatomy of organs from the middle abdominal region (sagittal section; 15 mm to the right of the median plane) (T2-weighted sequence) (Right kidney). Note: (CD) caudal; (CC) cranial; (D) dorsal; (V) ventral; (UB) urinary bladder; (p) renal pelvis; (h) renal hilus; (white line) renal medulla; (white star) renal cortex; (white point) corticomedullary junction; (S) stomach; (L) liver; (c) renal adipose capsule; (white cross) fibrous capsule; (oblique white arrowhead) renal recesses; (J) jejunum; (TC) transverse colon; (TD) transverse duodenum; (C) caecum; (PB) pubic bone; (I) ilium; (lm) longissimus muscle; (gl) gluteal muscle; (white perpendicular arrow) abdominal wall. Line = 10 mm.

The T2-weighted sagittal image of the abdominal organs 15 mm to the right of the median plane showed the characteristic findings of the right kidney and the urinary bladder. The adipose and fibrous capsules of the kidney were clearly distinguished. The corticomedullary junction, although well delineated, had irregular outlines. The inner and outer areas of the kidney medulla had signals of different intensities: hypointense-outer and hyperintense-inner medullary parts. The most intense signal characteristics were exposed by the renal pelvis. The image of the inner medulla was relatively hyperintense, separated radially by hypointense linear findings. The cranial border of the right kidney touched the liver. The right ureter was not visualized. The urinary bladder signal was the most intense (Figure 9).

**Figure 10:**
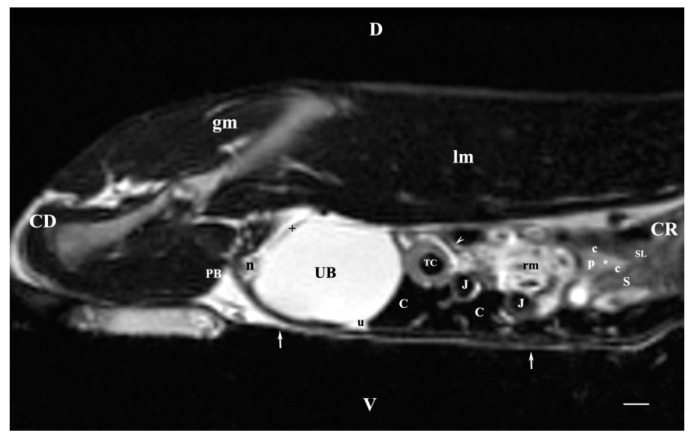
MRI imaging anatomy of organs from the middle abdominal region (sagittal section; 45 mm to the left of the median plane). (T2-weighted sequence) (Left kidney). Note: (CD) caudal; (CC) cranial; (D) dorsal; (V) ventral; (UB) urinary bladder; (p) renal pelvis; (c) renal adipose capsule; (white star) renal cortex; (n) neck of the urinary bladder; (oblique white arrowhead) ureter; (S) stomach; (SL) spleen; (J) jejunum; (TC) transverse colon; (C) caecum; (rm) mesenterial root; (PB) pubic bone; (u) urachus and median vesical ligament; (lm) longissimus muscle; (gm) gluteal muscle; (white perpendicular arrow) abdominal wall. Line = 10 mm.

The T2-weighted sagittal image of the abdominal organs 45 mm to the left of the median plane visualized the images of the lateral part of the left kidney, whereas the same section on the right side did not display any kidney signal. The left kidney was presented as a small, heterogeneous, relatively hyperintense finding with poorly delineated pelvis and parenchymal parts. The kidney was located caudoventrally to the spleen and the stomach. A segment of the hyperintense left ureter appeared in the dorsal part of the abdomen, caudal to the mesenterial root, and caudodorsal to the transverse colon. The signals of the urinary bladder apex, urachal remnant and body were of high intensity compared to the hypointense caudally located bladder neck. The bladder wall appeared as a hypointense band of irregular borders (Figure 10).

### 3.3. Dorsal MRI

**Figure 11:**
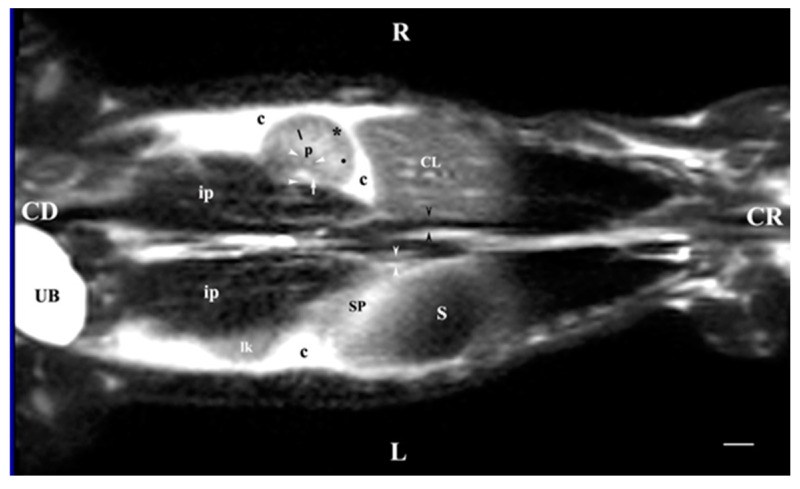
MRI imaging anatomy of organs from the middle abdominal region (dorsal section; 15 mm ventral to the vertebral column). (T2-weighted sequence). Note: (R) right; (L) left; (CR) cranial; (CD) caudal; (UB) urinary bladder; (p) renal pelvis; (perpendicular white arrow) commencement of right ureter; (oblique white arrowhead) dorsal renal recesses; (black star) renal cortex; (black line) renal medulla; (black point) corticomedullary junction; (lk) left kidney; (c) renal adipose capsule; (horizontal white arrowhead) renal hilus; (ip) iliopsoas muscle; (CL) caudate lobe; (S) stomach; (SP) spleen; (black perpendicular arrowhead) caudal vena cava; (white perpendicular arrowhead) abdominal aorta. Line = 10 mm.

The T2-weighted image of the abdominal cavity (dorsal section; 15 mm ventral to the vertebral column) demonstrated the intense heterogeneous signal of the right kidney along with the hypointense dorsal recesses. Only the dorsal part of the left renal cortex was visible. The signal of the adipose capsule was the most intense, marking the anatomical contrast between the right kidney and the caudate lobe of the liver and between the left kidney and the spleen. Medial to both kidneys appeared the hypointense heterogeneous images of both iliopsoas muscles. The hyperintense longitudinally oval urinary bladder remained in the caudal left abdomen (Figure 11).

**Figure 12:**
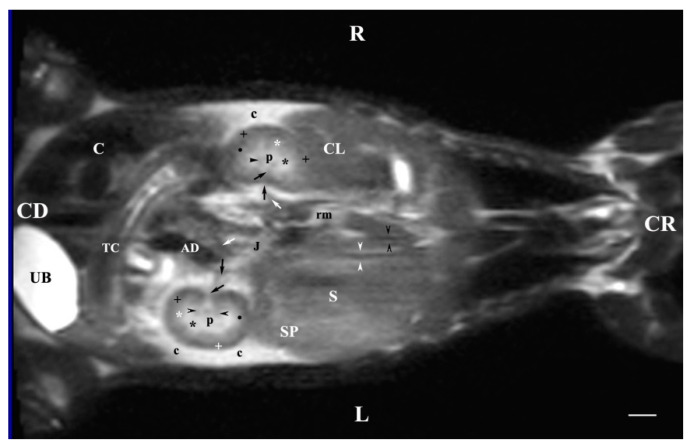
MRI imaging anatomy of organs from the middle abdominal region (dorsal section; 30 mm ventral to the vertebral column). (T2-weighted sequence). Note: (R) right; (L) left; (CR) cranial; (CD) caudal; (p) renal pelvis; (UB) urinary bladder; (black cross) renal cortex; (black star) inner part of the renal medulla; (white star) external part of the renal medulla; (perpendicular black arrow) renal hilus; (horizontal black arrowhead) transition between the dorsal and ventral recess; (oblique black arrow) renal sinus (c) renal adipose capsule; (black point) corticomedullary junction; (oblique white arrow) ureter; (CL) caudate lobe of the liver; (S) stomach; (SP) spleen; (black perpendicular arrowhead) caudal vena cava; (white perpendicular arrowhead) abdominal aorta; (C) caecum; (AD) ascending duodenum; (TC) transverse colon; (J) jejunum; (rm) root of mesentery. Line = 10 mm.

The T2-weighted dorsal image of the abdominal organs 30 mm ventral to the vertebral column demonstrated the image of the entire organs. The heterogeneous hyperintense right kidney was cranial to the image of the left one. The right kidney remained close to the median plane and the tubular signals of the caudal vena cava and the abdominal aorta, whereas the left kidney was visualized relatively laterally. Renal hiluses and the entry of both ureters appeared as hypointense findings at the medial renal border. The adipose capsule was well-defined and hyperintense compared to the barely visible hypointense linear image of the fibrous capsule. The two medullary areas were visible with specifically different signal intensities. The corticomedullary definition was observed as a transition between the hyperintense medulla and the relatively hypointense cortex. The transition between the dorsal and ventral recesses demonstrated a hypointense image. The renal pelvis signal was more intense as compared to the other renal structures. The contact between the right kidney and the caudate lobe of the liver was visualized. The contact between the left kidney and spleen was displayed. The urinary bladder remained in the left caudal abdomen and presented as a hyperintense longitudinally oval image (Figure 12).

**Figure 13:**
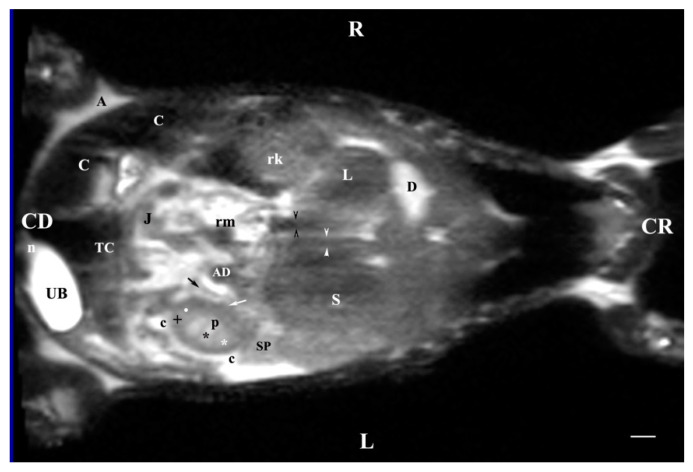
MRI imaging anatomy of organs from the middle abdominal region (dorsal section; 45 mm ventral to the vertebral column) (T2-weighted sequence). Note: (R) right; (L) left; (CR) cranial; (CD) caudal; (UB) urinary bladder; (p) renal pelvis; (rk) right kidney; (L) liver; (D) diaphragm; (S) stomach; (SP) spleen; (n) neck of the urinary bladder; (C) caecum; (TC) transverse colon; (AD) ascending duodenum; (J) jejunum; (rm) mesenterial root; (black oblique arrow) ureter; (white oblique arrow) renal hilus and ventral recess; (white star) outer part of the renal medulla; (black star) internal part of the renal medulla; (white point) corticomedullary junction; (black cross) renal cortex; (c) renal adipose capsule; (A) adipose tissue. Line = 10 mm.

The dorsal section of the T2-weighted images of the studied abdominal organs (45 mm ventral to the vertebral column) presented the entire left kidney. It was in contact with the spleen craniolaterally, whereas the stomach was craniomedial to the left kidney. The renal cortex signal was less intense than that of the medulla. It was with a distinct outer relatively hypointense part and hyperintense-inner part. The medullary image was heterogeneous due to the radial hypointense findings. The renal pelvis, the corticomedullary junction, the adipose and the fibrous capsule were visualized. The hypointense left renal hilus was located in the craniomedial part of the kidney. The ventral recessus was hypointense. The entry of the left ureter appeared caudomedial to the left kidney. Close to (medially) the kidney was the hypointense signal of the ascending duodenum. The abdominal aorta remained medially to the left kidney. Only the ventral heterogeneous intense signal of the right kidney that touched the liver cranially was displayed. The hyperintense adipose and hypointense fibrous capsules of the right kidney were scarcely presented, and medially to the kidney appeared the caudal vena cava. Caudal to both kidneys, the mesenterial root, parts of the jejunum and the transverse colon were found. The urinary bladder was the most intense, located in the caudoventral left part of the abdominal cavity. Its shape was elongated and oval; the large caecum was to its right side. The bladder neck signal was hypointense in relation to that of the bladder body (Figure 13).

**Figure 14:**
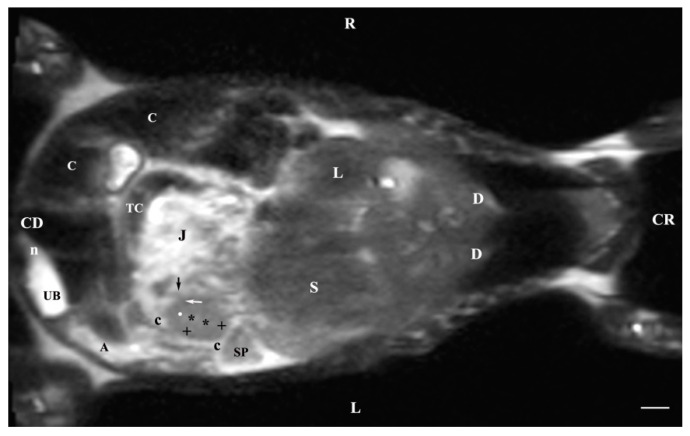
MRI imaging anatomy of organs from the middle abdominal region (dorsal section; 60 mm ventral to the vertebral column). (T2-weighted sequence). Note: (R) right; (L) left; (CR) cranial; (CD) caudal; (UB) urinary bladder; (n) neck of the urinary bladder; (black cross) renal cortex; (black star) renal medulla; (white point) corticomedullary junction; (L) liver; (D) diaphragm; (S) stomach; (SP) spleen; (c) renal adipose capsule; (black perpendicular arrow) left ureter; (white horizontal arrow) renal hilus; (J) jejunum; (TC) transverse colon; (C) caecum; (A) adipose tissue. Line = 10 mm.

The T2-weighted images of the kidneys, ureters and the urinary bladder (dorsal section; 60 mm ventral to the spine) demonstrated a relatively hypointense finding of the ventral left kidney; the right one presented no finding. The hyperintense adipose capsule and the hypointense fibrous capsule were scarcely defined. The left hilus and ureter appeared as hypointense luminal structures, caudomedial to the kidney parenchyma. There were no signals from the renal pelvis and renal medulla. The hyperintense image of the urinary bladder visualized its ventral part, along with the hypointense bladder neck (Figure 14).

## 4. Discussion

The present MRI study provides information about kidneys and ureters, allowing for the anatomic imaging resolution of organs. Similarly to the MRI study in humans, the animals were in dorsal recumbency with flexed stifle joints. The MRI scans were performed in the transverse, sagittal and dorsal planes. The normal kidney fibrous and adipose capsules in rabbits were defined relatively easily, unlike humans, where the fibrous capsule was visualized significantly better in kidney parenchyma inflammation [17,18,26].

Our MRI results, which demonstrate the topographic features of the rabbit kidneys (in the lumbar region of the abdominal cavity), correspond to the previously published data. Additionally, we obtained realistic results for the cranial position of the right kidney to the left kidney. The localization of the rabbit’s right kidney in the diapason from L1 to L3 and that of the left kidney from L2 to L4 differs from the description for the same organs in dogs. Otherwise, the topography of the rabbit kidney coincides with that of the feline kidneys [38,39,40].

The present study provides MRI anatomical data for the position of the urinary bladder in the rabbit without considering the gender or body weight of the studied animals. Thus, our protocol of investigation goals to present qualitative MRI findings without quantitative analysis is different from the algorithm of the radiologic study presented by some authors [38].

The predisposition of the rabbit urinary system to many diseases motivated us to conduct this MRI investigation. These results are useful for the interpretation of many urinary system diseases, as described for carnivores and humans [38,41].

On the T2-weighted sequences, the distinction between the renal cortex and medulla was more definitive, contrary to the previously published data [24]. The kidney fibrous capsule appeared as a thin linear hypointense finding, in line with reports for men [24].

The present study yielded information in support of the fact that the contrast on T2-weighted MRI images of rabbit kidneys is anatomical, similar to that of the T1-weighted sequences in chinchillas [40]. The intensity of the observed kidney findings was heterogeneous compared to the peripheral soft tissues [40]. The right kidney was visualized as a finding of soft tissue intensity, touching the hypointense liver and appearing cranially. The left kidney image was ventrocaudal and less well-defined vs. the adjacent soft tissues than the right one, contrary to what was reported for chinchillas [40]. On the T2-weighted MRI sequences, the shape of the kidneys of New Zealand White rabbits was similar to that of chinchillas [7,8,9,10,11,12,13,14,15,16,17,18,19,20,21,22,23,24,25,26,27,28,29,30,31,32,33,34,35,36,37,38,39,40]. The observed hypointense findings within the kidney medulla presented a pseudopapillary pattern of the rabbit pyelocaliceal system as compared to the multipapillary pattern of the renal parenchyma of the chinchilla’s kidney cortex [40].

The obtained results confirmed the hypothesis of the bilateral topography of rabbit kidneys, convincingly demonstrating their retroperitoneal localization [38]. The caudoventral location of the left kidney, found by the performed MRI scans, presented the left kidney as more mobile [38]. Similarly to findings in dogs [24], the right kidney of rabbits was cranially located [38]. Unlike cats, both kidneys of rabbits did not show bilateral symmetry [38]. This study was designed to present the image MR characteristics of the studied rabbit organs and not their morphometric features [38].

From the two existing MR urography protocols—static and dynamic [36]—our study applied the static one in order to use T2-weighted sequences with a long relaxation time of urine. This allowed for obtaining hyperintense images of the urinary tract and renal parenchymal structures, which were with a higher degree of hydration. The investigation of the kidneys, ureters and urinary bladder in the rabbit does not require a prior application of contrast; therefore, imaging is not dependent on the excretory phase in the dynamic protocol and depends only on the presence of urine in the collecting system and ureters [27,28].

The urinary bladder of the rabbit has an elongated neck, which is why the MR image of this organ was detected in the caudal part of the abdominal cavity. The renal pelvis of the rabbit was hyperintense, as its peripheral recesses were hypointense, unlike the findings reported for dogs [38]. The right and left kidney pelvis shapes differed from the specific features described in dogs [38].

On sagittal T2-weighted MRI (15 mm right to the median plane), the characteristics of the right kidney were similar to those of carnivores, as far as the hypointense linear radial findings in the inner medulla were concerned. Therefore, it may be affirmed that the hyperintense image of the renal papilla of rabbits is divided into pseudopapillary parts, similar to the kidneys of carnivores and small ruminants [42]. Probably, this is an image of the distal continuation of the outer medullary area and the cortex in the direction of the renal pelvis. The rabbit renal pelvis demonstrates a hypointense image of the dorsal and ventral recesses, like carnivores, in which the dorsal and ventral recesses are separated by distal continuations of the renal parenchyma and the interlobar vessels passing in this region [42]. Therefore, the results from sagittal MRI proved that the kidney medullary section of rabbits has a pseudopapillary pattern.

In our opinion, a good definition of the corticomedullary junction is a sign of intactness of the renal findings.

MRI of the normal rabbit kidney successfully visualized the corticomedullary junction on a T2-weighted sequence, with a relatively hypointense image of the kidney cortex vs. that of the kidney medulla due to the larger amount of extracellular medullary fluid. Thus, the obtained T2-weighted images were of high informative value and definitive. In our opinion, the lack of corticomedullary differentiation on the T2-weighted scans was a non-specific sign of kidney anomaly, unlike the T1-weighted images [16].

Similarly, to the use of rats as a biological model for investigation of the human kidneys, we suggest rabbits as an appropriate model because the hydration status of rabbits also influenced the intensity of the MR signal of the renal cortical and medullary parts. Similar to rats, rabbits possess two differentiated kidney medullary parts of various signal intensities. However, in rabbits, contrary to rats, the outer medullary area produces a signal of lower intensity compared to the inner area on T2-weighted scans [16]. Therefore, the hyperintense signal of the outer medulla is due to the structure, blood supply and reduced amount of interstitial fluid. The inner medullary area showed a higher signal intensity on the T2-weighted sequence because of the localization of distal straight tubules and collecting ducts, which results in the hydration of the area [16]. We believe that the definitive image of the renal corticomedullary junction on the T2-weighted MR scans is a reliable marker for the normal function of the organ.

This MRI study added to the application of the method for obtaining anatomical images of the kidneys and the topographical relationships of their different macroscopically structural parts (cortex, medulla and sinus). MRI is a definitive and informative method of higher grade, allowing quality and contrast imaging of the urinary tract organs [9,10,11,12].

The results from the performed MRI study supported the assumption that the rabbit is an appropriate animal model for kidney research [8,13,14,15].

Contrary to the theory that the rabbit kidney is unipapillary, the present study demonstrated image morphological traits that define the kidney of the studied animal species as pseudopapillary because of the distinction of radial hypo- and hyperintense areas in the medullary area [1]. The presence of images of the dorsal and ventral kidney recesses was confirmed [1]. Our study provided detailed MR imaging data in support of the thesis about the cranial localization of the right kidney [1]. The adipose capsule of the right kidney was more developed than that of the left one but did not cause ventral displacement of the kidney borders [1].

Due to the fact that kidney diseases are often encountered in rabbits: kidney agenesis, asymptomatic congenital renal cysts, bacterial infections, staphylococcal nephritis, pyelonephritis, renal amyloidosis, urolithiasis and neoplasms, we presented detailed and informative data on MRI-specific features of normal urinary organs [5,6,7]. Dissimilar to previous studies [5,6,7], reporting that only one of the kidneys in healthy rabbits was available for examination due to anatomical limitations, we succeeded in achieving informative MR access to both kidneys.

The results from the MRI examination of the kidney, ureters and urinary bladder in the rabbit enrich the morphological imaging knowledge about the diseases and abnormalities of these organs, such as inflammation, hydronephrosis, compensatory hypertrophy, infarction, dysplasia, hypoplasia, cysts, ectopy, reflux and neoplastic lesions and hydroureter, as well as urethral urolithiasis, resulting in an increased urinary bladder size [38,41,43].

The performed MRI study added to what was already reported [19,20,21,22,23]: that MR urography is a contemporary diagnostic imaging method for the morphological study of the whole urinary tract, which may replace excretory urography as a gold standard for imaging of urinary organs’ pathological states.

## 5. Conclusions

MRI is an innovative imaging modality for studying a rabbit’s urinary system. The obtained MRI results are of high quality and very informative because, in three anatomical aspects, data were obtained for the morphologic specifics of rabbit kidneys (the presence of corticomedullary differentiation, dorsal and ventral renal recesses and the visualization of the renal pelvis), ureters and the urinary bladder. This detailed information could be successfully applied as a base for the interpretation of many nephrology and urologic diseases, which are specific to rabbits as lagomorphs and other animals. At the same time, MRI is a conclusive method for providing detailed information if any other imaging modalities are insufficient.

## Data Availability

The datasets used during the current study are available from the corresponding author on reasonable request.
